# Publisher Correction: Crystal structure of a lipin/Pah phosphatidic acid phosphatase

**DOI:** 10.1038/s41467-020-15509-0

**Published:** 2020-04-02

**Authors:** Valerie I. Khayyo, Reece M. Hoffmann, Huan Wang, Justin A. Bell, John E. Burke, Karen Reue, Michael V. Airola

**Affiliations:** 1 Department of Biochemistry and Cell Biology, Stony Brook University, Stony Brook, NY USA; 2 Department of Biochemistry and Microbiology, University of Victoria, Victoria, BC V8W 2Y2 Canada; 3 Department of Human Genetics, David Geffen School of Medicine, UCLA, Los Angeles, CA USA

**Keywords:** Hydrolases, Membrane lipids, Membrane proteins, Mass spectrometry, X-ray crystallography

Correction to: *Nature Communications* 10.1038/s41467-020-15124-z, published online 11 March 2020.

The original version of this article contained an error in the spelling of the author Michael V. Airola, which was incorrectly given as Michael V. V. Airola. This has now been corrected in the HTML version of the article. The PDF was correct at the time of publication.

