# Supplementary material for: Crystal structure of a lipin/Pah phosphatidic acid phosphatase
Source: Nat Commun. 2020 Mar 11;11:1309. doi: 10.1038/s41467-020-15124-z (PMC7066176; doi:10.1038/s41467-020-15124-z)
Supplement: Supplementary file 1 — Supplementary Information [file 41467_2020_15124_MOESM1_ESM.pdf]

Crystal structure of a lipin/Pah phosphatidic acid phosphatase

Khayyo et al

Supplementary Figures and Information

Supplementary Table 1. HDX Data Summary

Data Set	*Tt* Pah2	*Tt* Pah2 + membrane
HDX reaction details	$\%D_2O$ = 93%<br>$pH_{(rea)}$ = 6.5<br>Temp = 18°	$\%D_2O$ = 93%<br>$pH_{(rea)}$ = 6.5<br>Temp = 18°
HDX time course	3s, 30s, 300s, 3000s, 10000s	3s, 30s, 300s, 3000s, 10000s
HDX controls	N/A	N/A
Back-exchange	Corrected based on $\%D_2O$	Corrected based on $\%D_2O$
Number of peptides	143	143
Sequence coverage	98.8%	98.8%
Average peptide length / Redundancy	Length = 13.5<br>Redundancy = 6.0	Length = 13.5<br>Redundancy = 6.0
Replicates	3	3
Repeatability	Average StDev = 0.6%	Average StDev = 0.6%
Significant differences in HDX	>4% and >0.3 Da and unpaired t-test <0.01	>4% and >0.3 Da and unpaired t-test <0.01

Supplementary Table 2. Primers

Construct	Forward Primer	Reverse Primer
D146A	5'-CTGGTGATCTCTGCCGTGGACGGCACCGTGACCAAAT	5’-ATTTGGTCACGGTGCCGTCCACGGCAGAGATCACCAG
G79R	5'-ATGCTGCCGGAACGCGCGTGCTACTTCCCGGAACTGAA	5'-AGTAGCACGCGCGTTCCGGCAGCATCAGCATGAAAAC
G267R	5'-TTCGCGGGCTTCCGCAACCGTGACACCGACGCTAC	5'-TGTCACGGTTGCGGAAGCCCGCGAAGATCGGGTTAA
L103P	5'-CTTCCGCTATCCCCAAAAAATTCAACCTGAAAAACGGCTA	5'-TTGAATTTTTTGGGGATAGCGGAAGACGGACGCAG
Y306N	5'-GAAAAATCCTCCAACAAGAAAATCAACGAAAAAATCCAGG	5'-TGATTTTCTTGTTGGAGGATTTTTCCTGTTTACCCAG
S191L	5'-GGTTTACCTGCTTTCTCGCCCGCTGTACTTCTACAA	5'-AGCGGGCGAGAAAGCAGGTAAACCATTTTGTAACCGTTTTTC
R193H	5'-ACCTGTCTTCTCACCCGCTGTACTTCTACAACTACAC	5'-AAGTACAGCGGGTGAGAAGACAGGTAAACCATTTTGTAA
∆Helix	5'-GGCGACGGATCCATGTTCTCTGGTGTTGTTGACATCATCGTC	5'-GGCGACGCGGCCGCCTACGGCAGACGCGGGAACAGTTC
TtPah2 1-335 in pcDNA	5'-GGCGACGAATTCCCACCATGATCAACGGTATCAAAAACCTGTTC	5'-GCCGACGCGGCCGCCTTAGGCAGTTGTTTATTCTGCTGC
TtPah2 1-321 in pcDNA	5'-GGCGACGAATTCCCACCATGATCAACGGTATCAAAAACCTGTTC	5'-GGCGACGCGGCCGCCGGCAGACGCGGGAACAGTTCCTG
Mouse Lipin-2	5'-GGCGACGGATCCATGAATTATGTGGGCCAGCTGGCT	5'-GGCGACGCGGCCGCCTAAGCCAGGTCATCCAGGTCCAGGT
Mouse Lipin-2 Delta 94-627	5'-GAGGAGACTGAAGGGACGGCCTCATATAAGAAGTCT	5'-TGAGGCCGTCCCTTCAGTCTCCTCTACAAAGAAGGCT

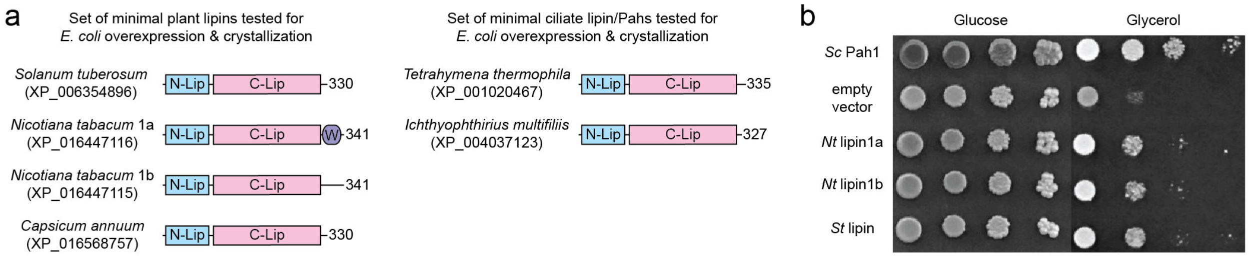

Supplementary Figure 1. Minimal lipin PAPs. (a) Six minimal lipin/Pah PAPs from plants or ciliates that directly fuse the N-Lip and C-Lip regions were cloned from cDNA (*S. tuberosum, N. tabacum*) or gene synthesized (*C. annuum, T. thermophila, I. multifiliis*). **(b)** Three minimal lipin/Pah PAPs were tested for complementation of *S. cerevisiae pah1*Δ cells to utilize glycerol as the sole carbon source. Spots represent 10-fold serial dilutions of *S. cerevisiae pah1*Δ cells containing the indicated plasmids.

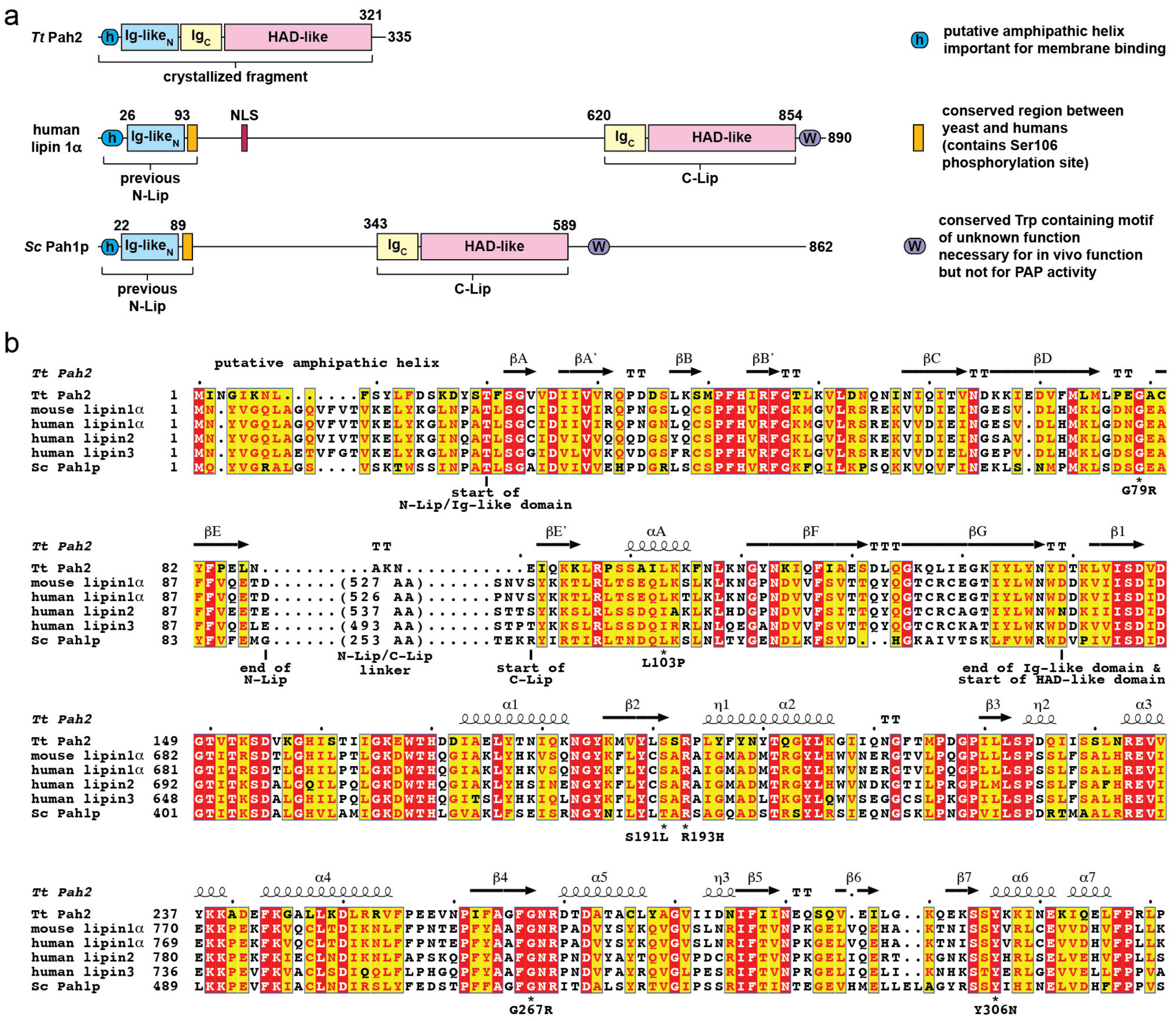

Supplementary Figure 2. Architecture and sequence alignment of *Tt* Pah2 with human and yeast PAPs. (a) Updated domain architecture of PAPs drawn to scale. Amphipathic helix (blue h), immunoglobulin-like domain divided into the N-Lip (Ig-like$_N$, light blue) and C-Lip (Ig$_C$, yellow) sections, haloalkanoic acid dehalognease-like (HAD-like) domain (pink), conserved region in lipin PAPs and *Sc* Pah1 previously annotated as part of the N-Lip (orange), conserved motif containing the Trp residue essential for in vivo function but not in vitro PAP activity (purple W). **(b)** Sequence alignment of *Tt* Pah2 with human and mice lipins and *Sc* Pah1. Identical residues shaded red, homologous residues shaded yellow. Secondary structure elements for the *Tt* Pah2 calcium structure are indicated above, “TT” = turn. Disease-associated missense mutations are indicated below each residue, labels refer to the residue number in *Tt* Pah2.

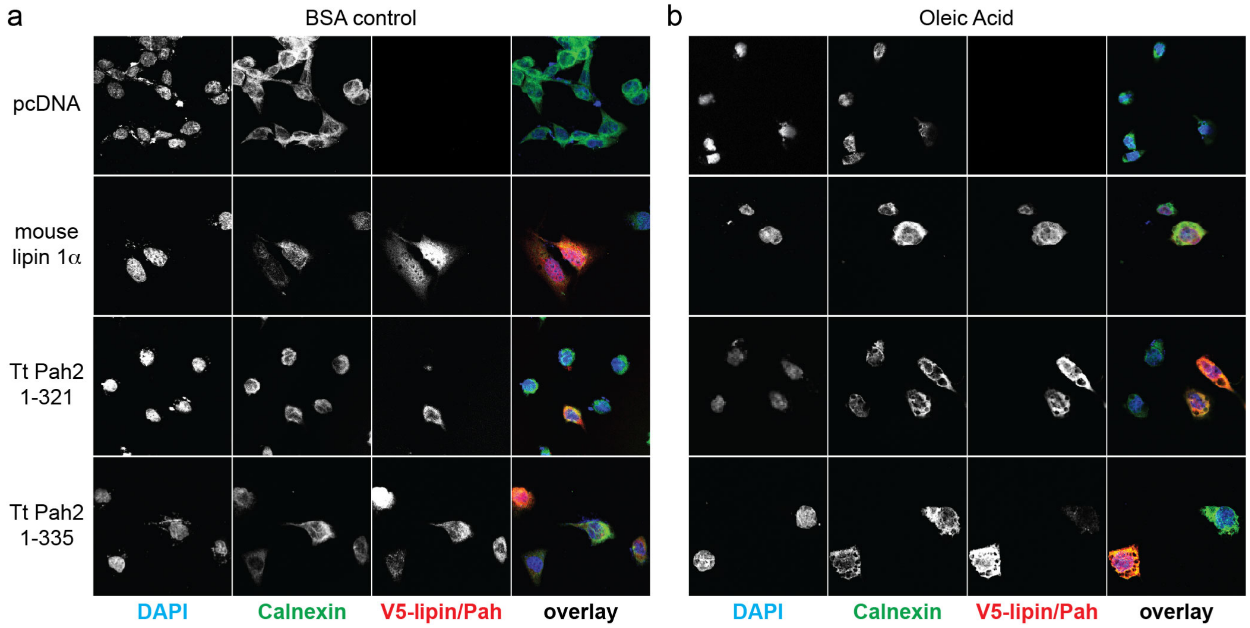

Supplementary Figure 3. Cellular localization of lipin/Pah PAPs in HEK293 cells. Confocal images of lipin/Pah constructs in HEK293 cells visualized by immunofluorescence. Cells were treated with **(a)** BSA control or **(b)** with oleic acid. DAPI indicates the cell nucleus, Calnexin indicates the endoplasmic reticulum (ER).

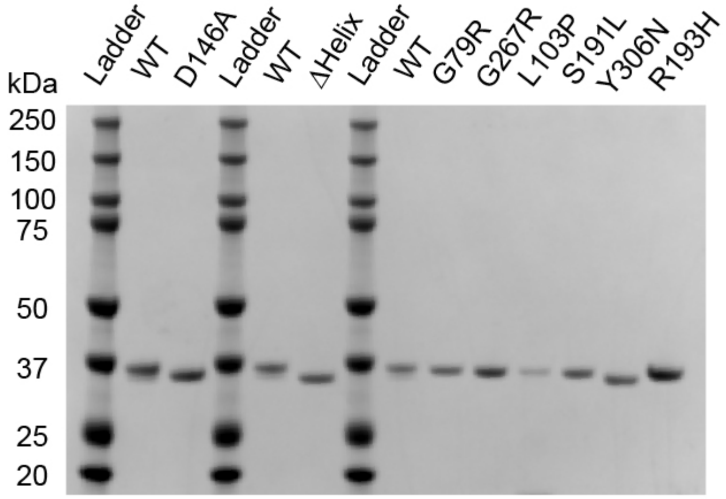

Supplementary Figure 4. SDS-PAGE analysis of purified wild-type (WT) *Tt* Pah2, the D146A DxDxT mutant, the ΔHelix construct, and disease-associated mutants used in this study. Approximately 2 μg of each protein was loaded, except for L103P due to low yields. Protein bands visualized by coomassie blue staining.

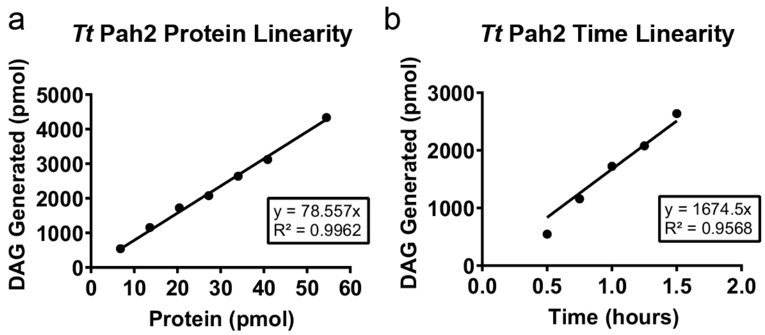

Supplementary Figure 5. Lipin PAP assay parameters. (a) *Tt* Pah2 activity assays were linear in respect to protein concentration between 7 – 54 pmol of *Tt* Pah2. Assays were performed in the linear range at a concentration with 27 pmol of *Tt* Pah2 with 10 mol% NBD-PA in Triton X-100 mixed micelles for 1 hr at 37 ˚C. **(b)** *Tt* Pah2 activity assays were linear in respect to time at 1 hr. Time linearity was determined by iterative 15-minute incubation and subsequent quenching with $MeOH/CHCl_3$.

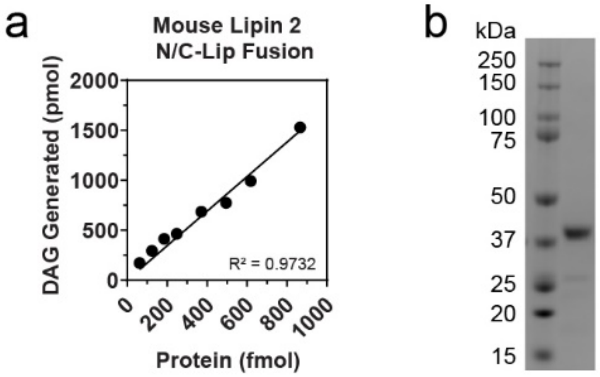

Supplementary Figure 6. Biochemical characterization of an N-Lip/C-Lip fusion of mouse lipin 2. (a) PAP activity of the mouse lipin 2 N-Lip/C-Lip fusion was linear in respect to protein concentration between 62 – 866 fmol. Assays were performed with 10 mol% NBD-PA in Triton X-100 mixed micelles for 1 hr at 37 ˚C. **(b)** SDS-PAGE analysis of the purified N-Lip/C-Lip fusion of mouse lipin 2. Approximately 2 µg of protein was loaded. Protein bands were visualized by coomassie blue staining.
