# Supplementary material for: Crystal structure of a lipin/Pah phosphatidic acid phosphatase
Source: Nat Commun. 2020 Mar 11;11:1309. doi: 10.1038/s41467-020-15124-z (PMC7066176; doi:10.1038/s41467-020-15124-z)
Supplement: Supplementary file 3 — Reporting Summary [file 41467_2020_15124_MOESM3_ESM.pdf]

nature research

Corresponding author(s): Michael Airola

Last updated by author(s): 02/18/2020

Reporting Summary

Nature Research wishes to improve the reproducibility of the work that we publish. This form provides structure for consistency and transparency in reporting. For further information on Nature Research policies, see Authors & Referees and the Editorial Policy Checklist .

Statistics

For all statistical analyses, confirm that the following items are present in the figure legend, table legend, main text, or Methods section.

n/a	Confirmed	
☐	☒	The exact sample size (*n*) for each experimental group/condition, given as a discrete number and unit of measurement
☐	☒	A statement on whether measurements were taken from distinct samples or whether the same sample was measured repeatedly
☒	☐	The statistical test(s) used AND whether they are one- or two-sided<br>*Only common tests should be described solely by name; describe more complex techniques in the Methods section.*
☒	☐	A description of all covariates tested
☒	☐	A description of any assumptions or corrections, such as tests of normality and adjustment for multiple comparisons
☒	☐	A full description of the statistical parameters including central tendency (e.g. means) or other basic estimates (e.g. regression coefficient) AND variation (e.g. standard deviation) or associated estimates of uncertainty (e.g. confidence intervals)
☒	☐	For null hypothesis testing, the test statistic (e.g. *F*, *t*, *r*) with confidence intervals, effect sizes, degrees of freedom and *P* value noted<br>*Give P values as exact values whenever suitable.*
☒	☐	For Bayesian analysis, information on the choice of priors and Markov chain Monte Carlo settings
☒	☐	For hierarchical and complex designs, identification of the appropriate level for tests and full reporting of outcomes
☒	☐	Estimates of effect sizes (e.g. Cohen's *d*, Pearson's *r*), indicating how they were calculated

Our web collection on statistics for biologists contains articles on many of the points above.

Software and code

Policy information about availability of computer code

Data collection	no software was used
Data analysis	Coot 0.8.9.1<br>Phenix 1.15rc1-3423<br>CCP4 7.0.000<br>GraphPad Prism 8.1

For manuscripts utilizing custom algorithms or software that are central to the research but not yet described in published literature, software must be made available to editors/reviewers. We strongly encourage code deposition in a community repository (e.g. GitHub). See the Nature Research guidelines for submitting code & software for further information.

Data

Policy information about availability of data

All manuscripts must include a data availability statement. This statement should provide the following information, where applicable:

- Accession codes, unique identifiers, or web links for publicly available datasets
- A list of figures that have associated raw data
- A description of any restrictions on data availability

Data availability statement. Coordinates and structure factors have been deposited in the Protein Data Bank under accession codes 6TZY and 6TZZ. The mass spectrometry proteomics data have been deposited to the ProteomeXchange Consortium via the PRIDE57 partner repository with the dataset identifier PXD017575. All other data are available from the authors on request. The source data underlying Figs 1c, 4a–e, 4g, 5c-f, and 6c are provided as a Source Data file.

Field-specific reporting

Please select the one below that is the best fit for your research. If you are not sure, read the appropriate sections before making your selection.

☒ Life sciences ☐ Behavioural & social sciences ☐ Ecological, evolutionary & environmental sciences

For a reference copy of the document with all sections, see nature.com/documents/nr-reporting-summary-flat.pdf

Life sciences study design

All studies must disclose on these points even when the disclosure is negative.

Sample size	Two or three independent experiments were conducted. Each experiment was performed in technical duplicates and experimental duplicates or triplicates.
Data exclusions	No data was excluded.
Replication	All replication efforts were successful. Each experiment was performed in technical duplicates and experimental duplicates or triplicates.
Randomization	Allocation was not randomized.
Blinding	Blinding was not possible for these experiments.

Reporting for specific materials, systems and methods

We require information from authors about some types of materials, experimental systems and methods used in many studies. Here, indicate whether each material, system or method listed is relevant to your study. If you are not sure if a list item applies to your research, read the appropriate section before selecting a response.

Materials & experimental systems

n/a	Involved in the study	
☐	☒	Antibodies
☐	☒	Eukaryotic cell lines
☒	☐	Palaeontology
☒	☐	Animals and other organisms
☒	☐	Human research participants
☒	☐	Clinical data

Methods

n/a	Involved in the study	
☒	☐	ChIP-seq
☒	☐	Flow cytometry
☒	☐	MRI-based neuroimaging

Antibodies

Antibodies used	mouse monoclonal V5 (Invitrogen, Cat. 960-25)<br>Calnexin (Abcam, Cat. ab22595)<br>Alexa 647-labeled donkey-anti-mouse (Invitrogen, Cat. A31571)<br>Alexa 488-labeled donkey-anti-rabbit (Invitrogen, Cat. A21206)
Validation	All of the antibodies used in this study are commercially-made antibodies and they have been tested and verified both by the manufacture and by our own experiments with appropriate positive and negative controls.<br><br>Specifically,<br>For Mouse V5 and Calnexin:<br>Western blot has been used to verify the specificity of antibodies with the correct sizes. Protein with V5 tag was used as a positive control.<br>For Alexa Fluor 647 and 488:<br>Negative control was used in IF experiment to ensure no cross-reaction between secondary antibody and samples.

Eukaryotic cell lines

Policy information about cell lines

Cell line source(s)	Human embryonic kidney 293 cells (HEK293, American Type Culture Collection #CRL-1573)

Authentication	HEK293 cell line was purchased from ATCC (CRL-1573). Cells were carefully handled and expanded right after arrival. Early cell passage number 2-5 was used to ensure prevent genetic drift and mycoplasma contamination developed after too many passages.
Mycoplasma contamination	Early cell passage number 2-5 was used to ensure prevent mycoplasma contamination after too many passages.
Commonly misidentified lines (See ICLAC register)	HEK293 cells were used to achieve high rates of transfection
